# Ten-Year Trends (1999–2010) of Adherence to the Mediterranean Diet among the Balearic Islands’ Adult Population

**DOI:** 10.3390/nu9070749

**Published:** 2017-07-14

**Authors:** Maria del Mar Bibiloni, Mónica González, Alicia Julibert, Isabel Llompart, Antoni Pons, Josep A. Tur

**Affiliations:** Research Group on Community Nutrition and Oxidative Stress, University of the Balearic Islands, and CIBEROBN CB12/03/30038, Guillem Colom Bldg Campus, E-07122 Palma de Mallorca, Spain; mar.bibiloni@uib.es (M.d.M.B.); monica.gonzalez@ssib.es (M.G.); aliciajulibert@gmail.com (A.J.); isabel.llompart@ssib.es (I.L.); antonipons@uib.es (A.P.)

**Keywords:** nutrition survey, Mediterranean dietary pattern, Balearic Islands, Mediterranean region, adults

## Abstract

The aim of this work was to assess ten-year trends (1999–2010) of adherence to the Mediterranean dietary pattern (MDP) among the Balearic Islands’ adult population. Two independent cross-sectional dietary surveys (1999–2000, *n* = 1200 and 2009–2010 *n* = 1388, including participants aged 16–65 years) were carried out in the Balearic Islands, Spain. Dietary habits were assessed by means of two 24 h diet recalls and a validated semi-quantitative food-frequency questionnaire that covers 145 food items. Adherence to the MDP was defined according to a score constructed considering the consumption of nine MDP characteristic components: high monounsaturated fatty acids:saturated fatty acids (MUFA:SFA) ratio, moderate ethanol consumption, high legumes, cereals and roots, fruits, vegetables, and fish consumption, and low consumption of meat and milk. Socio-economic status, education level, lifestyle factors and health status were also assessed. Adherence to the MDP was 43.1% (SD 5.8) in 1999–2000 and 44.6% (SD 8.3) in 2009–2010. Higher age was directly associated with higher adherence to the MDP, and this association was stronger in males than in females. Young generations and smokers showed the lowest adherence to MDP, whereas people with higher educational and socio-economic level, and who were physically active showed the highest adherence. According to the place of birth, the increase in the percentage of the adherence to the MDP was observed to be smaller among the Balearic Island’s natives than among people born abroad. In 2009–2010, individuals in the MDP’s fourth quartile were more likely to be older (aged 46–65 years), and were less likely to have a low occupational level, to have a light physical activity level and to be smoker than in 1999–2000. The adherence to the MDP has been stabilized and slightly recovered among the Balearic Islands’ adult population in the last decade.

## 1. Introduction

The traditional Mediterranean diet is characterised by a high intake of vegetables, legumes, fruits and nuts, and cereals (which in the past were largely unrefined), a high intake of olive oil but a low intake of saturated lipids. It is also characterised by a moderately high intake of fish (depending on the proximity of the sea), a low-to-moderate intake of dairy products (and then mostly in the form of cheese or yoghurt), a low intake of meat and poultry and a regular but moderate intake of ethanol, primarily in the form of wine and generally during meals [1]. The traditional Balearic diet corresponds to the typical Mediterranean dietary pattern (MDP) [2].

Ecological evidence suggesting beneficial health effects of the MDP emerged from the classic studies of Keys [1,3,4]. Overall, the MDP is considered a healthy prudent dietary pattern and a high adherence to it has been associated with a better health status, due to the protective effect that this pattern shows against various chronic diseases [5,6,7], including a favourable effect on total mortality, cardiovascular disease (CVD) and cancer [8,9,10,11,12]. Further, several countries bounded by the Mediterranean Sea are among the nations with higher life expectancy at birth in the world, with Spain, France, Italy, Greece, Cyprus and Israel showing a lifespan of 80 years [13]. The MDP has been proposed as one of the determinants of the longevity of these populations [14,15].

In spite of the increasing evidence and public knowledge of the health benefits of the MDP [7,16], MDP was reported as being gradually substituted by the “western” or “globalized” dietary patterns in countries of the Mediterranean littoral over recent times [17,18,19,20]. Epidemiological evidence suggests that dietary patterns in the Mediterranean countries are changing rapidly, with an increased consumption of animal products rich in saturated fat and a decline of intake of basic foodstuffs based on vegetables [20]. Reasons for this development can be found in the substantial socio-economic changes throughout all of Europe over the past 40 years. All the recent nutritional surveys carried out in Spain in different groups of the population confirm a progressive departure from the traditional Mediterranean diet, mainly in younger generations [20,21,22,23,24,25]. However, available data on the nutritional transition in Mediterranean countries are scanty and rather heterogeneous across different geographical areas [26,27,28,29,30,31]. Thus, the aim of this work was to assess ten-year trends (1999–2010) of adherence to the Mediterranean dietary pattern (MDP) among the Balearic Islands’ adult population.

## 2. Methods

### 2.1. Study Design

The present analysis was performed using a database of two population-based cross-sectional nutritional surveys carried out in the Balearic Islands, Spain: the “Nutritional Study of the Balearic Islands” (ENIB survey, 1999–2000) and the “Prevalence of Obesity in the Balearic Islands: relationship with oxidative stress and inflammatory mediators” study (OBEX survey, 2009–2010).

### 2.2. Selection of Participants, Recruitment and Approval

In both the ENIB and OBEX surveys, the target population consisted of all inhabitants living in the Balearic Islands aged 16–65 years, and the sample populations were derived from residents registered in the official population census of the Balearic Islands. The theoretical sample size was 1500 individuals for both surveys in order to provide a specific relative precision of 5% (type I error = 0.05; type II error = 0.10), taking into account an anticipated 70% participation rate. The sampling techniques included stratification according to geographical area and municipality size (six strata), age (three strata) and sex of inhabitants, and randomisation into subgroups. The Balearic Islands municipalities were the primary sampling units, with individuals within these municipalities comprising the final sample units. Target eligibility criteria were being an apparently healthy man or woman aged 16–65 years. Pregnant women were not considered in both studies. The final sample was 1200 individuals in the ENIB survey (1999–2000, 80% participation) and 1388 individuals in the OBEX survey (2009–2010, 93% participation). The reasons for non-participation were: (1) the subject declined to be interviewed and (2) involuntary non-participation due to census error caused by address changes. The present study was conducted according to the guidelines laid down in the Declaration of Helsinki, and all procedures involving human subjects were approved by the Balearic Islands Ethics Committee (approval reference number IB/2251/14-PI). Written informed consent was obtained from all subjects.

### 2.3. General Questionnaire

A questionnaire incorporating the following questions were used: sex, age, marital status, place of birth (defined as being born in the Balearic Islands, East of Spain as representative of the Spanish Mediterranean coast, other parts of Spain and other countries), educational level (grouped according to years and type of education: low, <6 years at school; medium, 6–12 years of education; high, >12 years of education), socio-economic status (based on the occupation and classified as low, medium and high, according to the Spanish Society of Epidemiology methodology [32]), and smoking habit (yes; occasionally, <1 cigarette/day; no). Physical activity (PA) was evaluated according to guidelines for data processing and analysis of the international physical activity questionnaire (IPAQ) [33] in the short form. The specific types of activity assessed were walking, moderate-intensity activities (i.e., PA at work), vigorous-intensity activities (i.e., sport practice) and sitting time (used as an indicator variable of time spent in sedentary activity). Information on hours per day of television viewing, computer use, video games, other leisure time physical activity practice and typical sleep duration to the nearest 10 min was also included. The physical activity assessed by the international physical activity questionnaire was correlated with physical activity level (PAL) according to the estimation of the individualized activity coefficient. Each subject was classified taking into account their PAL value [34] as: sedentary (PAL ≥ 1.0 < 1.4), light (PAL ≥ 1.4 < 1.6), active (PAL ≥ 1.6).

### 2.4. Dietary Questionnaire 

Dietary questionnaire included two 24 h diet recalls and a semi-quantitative food-frequency questionnaire (FFQ). The FFQ was previously validated [35] and covers 145 food items (118 of the original validated FFQ plus the most characteristic Balearic Islands foods in order to facilitate the interviewee answer), and arranged by food type. Frequency of food consumption was based on times that food items were consumed (frequency per day, week or month). Consumption < 1/month was considered no consumption. Daily consumption (g) was determined by dividing the reported amount of the intake by the frequency (day). The 24 h recall was carried out twice during the study period; the first in the warm season (May–September) and the second in the cold season (November–March). This was to avoid the influence of seasonal variations. To avoid bias brought on by day-to-day intake variability, the questionnaires were administered homogeneously from Monday to Sunday. Volumes and portion sizes were reported in natural units, household measures, or with the aid of a book of photographs [36]. Conversion of food into nutrients was made using a self-made computerised program based on Spanish [37,38] and European [39] food composition tables and complemented with food composition data available for Majorcan food items [40]. Information about food consumption patterns was obtained from the food frequency questionnaire, whereas information on nutrient intake was derived from the average food daily consumption reported in the two 24 h recalls [30]. Identification of under-reported food intake was made using the energy intake:BMR (Basal Metabolic Rate) ratio; <1.14 classified the individual as an under-reporter [41]. 

### 2.5. Mediterranean Dietary Pattern

The MDP was defined according to a previously defined score indicating the degree of adherence to the traditional Mediterranean diet [1,23,30]. This Mediterranean dietary score (MDS) was converted to relative percentage of adherence using a previously described method [23,30] that will now be briefly summarised. 

An energy-adjusted value was obtained for each individual for the daily consumption of legumes, cereals and roots (including bread and potatoes), fruit (including nuts), vegetables, fish, meat (and meat products) and whole milk (and whole milk products). In order to score “moderate alcohol consumption”, a transformation centred at the level of consuming 30 g/day for men (30–(30–absolute alcohol intake)), and 20 g/day for women (20–(20–absolute alcohol intake)) was used to obtain the highest value for men consuming 30 g/day or women consuming 20 g/day, and progressive lower values as the consumption was lower or higher than these values. These values were associated with the lowest CHD (coronary heart disease) risk in previous studies [42,43]. Information about the consumption of all these food items was obtained from the FFQ. The daily intake of fatty acids was calculated as the average intake recorded from the two 24 h recalls, and the MUFA: saturated fatty acids (SFA) ratio was calculated.

All these values were standardised as a *Z* value. A *Z* score expresses the difference between the individual’s measurement and the mean value of the reference population (in this case, the study population), as a proportion of the SD of the reference population (observed intake–mean intake/SD). The total MDS was computed by adding up all the *Z* scores obtained for the favourable or more Mediterranean dietary components (legumes, cereals and roots, fruit, vegetables, fish, moderate alcohol, MUFA:SFA ratio) and subtracting the *Z* value obtained from the consumption of meat and whole milk (mainly high in fat):

∑*Z*_i_ = *Z*_legumes_ + *Z*_cereals and roots_ + *Z*_fruit_ + *Z*_vegetables_ + *Z*_fish_ + *Z*_moderate alcohol_ + *Z*_MUFA:SFA_ − *Z*_meat_ − *Z*_milk_

The MDS was converted to relative percentage of adherence using the range of values of the sample. This percentage ranged from 100 (maximum adherence) and 0 (minimum adherence):

Adherence (Percentage_i_) = (∑*Z*_i_ − ∑*Z*_min_) × 100/(∑*Z*_max_ − ∑*Z*_min_)

Once the percentage of adherence to the MDP was calculated, the variables that could determine the highest adherence to the MDP (quartile 4) were assessed. The highest adherent participants to the MDP were defined as a score ≥45.3% in the period of interview 1999–2000 and ≥48.6% in the period of interview 2009–2010.

## 3. Statistics

Analyses were performed with SPSS version 24.0 (IBM SPSS Statistics, Nairobi, Kenya). Mean adherence values and SD were calculated. Statistical analysis was performed by ANCOVA, trend analysis, adjusted by sex and age. It was applied to compare values of adherence to the Mediterranean diet in the different areas for both periods and to assess the association between sociodemographic and lifestyle variables with respect to the period of interview. The differences in prevalence across high adherence to the MDP (fourth quartile) and other participants (quartiles 1 + 2 + 3) according to socio-demographic and lifestyle variables in each period of interview were examined by using χ^2^ test. Binary logistic regression analyses were used to compare the period of interview (independent variable, 1999–2000 as reference category) between high adherent participants to the MDP (fourth quartile) and the other participants as reference value (quartiles 1 + 2 + 3) (dependent variable) in selected socio-demographic and lifestyle variables. The level of significance was established for *p* values < 0.05.

## 4. Results

The mean adherence was 43.1% (SD 5.8) and the median adherence was 42.1% in the period of interview 1999–2000, and the mean adherence in the period of interview 2009–2010 was 44.6% (SD 8.3) and the median adherence was 43.7% (*p* < 0.001). 

Table 1 shows the percentage of highest adherent individuals (i.e., individuals within the MDP’s fourth quartile) according to selected socio-demographic and lifestyle variables in each period of interview. There were statistically significant differences in socio-economic level and physical activity level variables in the period of interview 1999–2000; and in age group, marital status, place of birth, physical activity levels and smoking habit variables in the period of interview 2009–2010. Table 2 shows the mean and SD of the percentage of adherence to the MDP according to period of interview and selected socio-demographic and lifestyle variables. The adults studied showed similar adherence score to the MDP in the following groups: 16–25 years, divorced, widowed and single, low educational level, low and high occupational level, physical activity levels (sedentary, light, and active), and smoking habits (yes, occasionally, and no). There were significant differences in sex, age (26–45 and 46–65 years), married, place of birth, educational level (medium and high level) and socio-economic status (medium occupational level). In the period of interview 2009–2010, highest adherent individuals were more likely to be older (aged 46–65 years), and were less likely to have a low occupational level, to have a light physical activity level and to be smoker in comparison with the period of interview 1999–2000 (Table 3). 

## 5. Discussion

The main finding of the present study is that the adherence to the MDP has been stabilized and slightly recovered among the Balearic Islands’ population in the last decade, in the same sense of other Spanish findings [44]. In nutritional epidemiology, analyses of individual nutrients and food often ignore important interactions between components of a diet. For this reason, there is an increasing interest in the study of dietary patterns because individuals do not eat isolated nutrients. The MDP could offer an interesting alternative in health promotion because this pattern, and not only its individual nutrients, has been postulated as being protective against several diseases [5,6,7,8,9,10]. Nevertheless, despite all the increasing evidence about the benefits of the MDP, nowadays we are witnessing a reduction in the quality of this diet in many Mediterranean regions towards a more Western form [17,18,19,20]. A major shift from the traditional Mediterranean diet has occurred before the period covered by our study [3,17,19,45,46]; and a nutrition transition to a diet with lower intake of cereals and higher consumption of meat and dairy products has been reported in the Mediterranean region since the 1990s [24,47].

Higher age was directly associated with a higher adherence to the MDP. It is not surprising that older age group subjects had more health-conscious dietary habits than young adults (that is, higher consumption of fruit and vegetables, lower consumption of meats), as the former are more prone to several health conditions known to be associated to diet. Previous studies on dietary pattern assessment carried out in Spain also found an association between age and a greater adherence to the MDP [22,23,48]. These findings have also been replicated in other Mediterranean regions such as Italy and Greece [49,50]. It is probable that older individuals, with risk factors, had changed their diet in a healthier way as a result of medical advice or prescription, as it has been observed in previous studies [51]. When we analyzed the influence of age separately by sex, we could observe that this trend was more pronounced in males than in females (data not shown), in both periods. There were significant differences between the two periods in the age groups 26–45 and 46–65 years, being higher in the period 2009–2010. In contrast to younger ages, the difference was not statistically significant. This confirms that the younger generations continue at high nutritional risk, and it has also been demonstrated that the Mediterranean diet contributes to nutritional quality in Spanish schoolchildren and adolescents [52,53].

Socio-economic status and educational levels were important determinants of the health status in a community. There has been some speculation as to which dietary factors contribute to disease inequalities [54,55]. In the present study, we found significant differences in the percentage of adherence to the MDP, between the two periods, finding a greater percentage of adherence to high educational levels and socio-economic in 2009–2010. In previous studies carried out in Europe, socioeconomic indicators were also associated with higher adherence to the MDP [56,57]; however, other studies did not find this association [58,59]. Previous studies carried out in Spain did not find variations in the adherence to the Mediterranean diet according to educational level or social class [22,25]. However, we have previously found that lower socio-economic and educational groups were at high risk of low intake of antioxidant rich foods [60], and these trends are also present within the studied decade. Also, studies conducted in Spain have demonstrated that low socioeconomic and educational levels had low consumption of fruits and vegetables but high consumption of legumes and cereals [22,23,24,25,61]. Similarly, Darmon et al. [62] showed that education and other socioeconomic indexes are related to diet quality. In addition, there was higher adherence in the married group too. So, these results agree with previous studies, which reported a higher adherence to the MDP among subjects who were married/cohabiting [63,64].

A more physically active lifestyle was associated with a greater adherence to the Mediterranean diet. This association has been observed in other studies [22,25,49,51]. Thus, it seems that healthy lifestyle variables tend to cluster and therefore we could speak about the Mediterranean lifestyle, including a higher level of physical activity as one aspect associated with the MDP that can be protective against different diseases. Our results indicated that there was higher adherence if there was more physical activity, but comparing the two periods of interview the adhesion was maintained.

Surprisingly, in the period of interview 1999–2000 we could also observe in the analysis that smoking was also associated with a greater adherence to the Mediterranean diet. It has been documented that smoking is linked to less-healthy dietary habits that might contribute to the higher risk for cancer and CVD in smokers [65,66]. This result should be interpreted with caution. This association has been carefully analyzed and we have observed that this was not replicated when under-reporters were included in the analysis. The diet of smokers was also further analyzed and it was not significantly different to that of non-smokers, but it was slightly more fatty and sweeter than the non-smokers’ diet. Therefore, it is possible that the association was caused by chance. Also by taking into account the related power reduction and lower precision due to the decrease in the sample size of smokers after excluding under-reporters, this association would not represent the real situation that may exist in the reference population. In the period of interview 2009–2010, smokers are observed to have a lower adherence to the MDP compared to the smokers of the period 1999–2000, and between the populations in the two periods studied, no statistically significant differences were shown.

Based on the place of birth, we observed that the adherence to the MDP was significantly higher for the 2009–2010 period in all four region categories than for the 1999–2000 period. In particular, this increase in the percentage of the adherence to the MDP was observed to be smaller among the Balearic Island’s natives than among people born abroad.

Nevertheless, the present study has several limitations which should be taken into consideration when interpreting its findings. Firstly, the cross-sectional designs provided no basis for studying causality. Longitudinal data would provide a more valid and reliable estimate of the adherence to the MDP. Secondly, dietary and physical activity data were based on self-reports.

## 6. Conclusions

The present study demonstrated that the adherence to the MDP has been stabilized and slightly recovered among the Balearic Islands’ adult population in the last decade. Higher age was directly associated with high adherence to the MDP and this association was higher in males than in females. Younger generations and smokers showed low adherence to the MDP, whereas people with higher educational and socio-economic level, and who were physically active showed higher adherence. Therefore, the promotion of not only the MDP but also the Mediterranean lifestyle, including greater physical activity, should be reinforced in the Balearic younger generations. According to the place of birth, the increase in the percentage of the adherence to the MDP was observed to be smaller among the Balearic Islands’ natives than among people born abroad, in the last ten years. 

## Figures and Tables

**Table 1:** Percentage of high adherent participants to the Mediterranean dietary pattern (MDP) (quartile 4) according to period of interview and selected socio-demographic and lifestyle variables.

Socio-Demographic and Lifestyle Variables	Period of Interview (2009–2010)	Period of Interview (1999–2000)
Sex		
Male	27.6	19.9
Female	23.1	26.2
*p*-value	0.118	0.120
Age group		
16–25	18.4	23.4
26–45	25.5	22.9
46–65	39.6	28.6
*p*-value	0.001	0.276
Marital status		
Divorced	30.2	30.3
Widowed	30.0	27.8
Married	30.6	25.4
Single	0.028	0.811
*p*-value		
Place of birth		
Balearic Islands	21.0	23.1
Spanish East coast	38.6	34.0
Other Spanish regions	33.3	30.2
Other country	30.6	20.6
*p*-value	0.001	0.156
Educational level		
Low	26.3	28.0
Medium	22.9	26.6
High	28.5	21.5
*p*-value	0.168	0.532
Socio-economic status		
Low occupational level	19.8	31.8
Medium occupational level	27.7	21.4
High occupational level	27.8	32.5
*p*-value	0.058	0.023
Physical activity levels		
Sedentary	20.2	26.1
Light	27.4	42.7
Active	28.0	25.0
*p*-value	0.032	0.016
Smoking habit		
Yes	18.1	48.9
Occasionally	32.7	32.5
No	26.7	42.9
*p*-value	0.014	0.091

Educational level is grouped according to years and type of education: low (6 years of education); medium (6–12 years of education); high (>12 years of education). Socio-economic level is based on the occupation of the head of household. Physical activity level was determined according to the level of exercise reported by the interviewee during their free time. Smoking habit was classified as: no (never); yes (>1 cigarette/day); occasionally (<1 cigarette/day). Data are expressed as percentage (%) of row; statistical analysis was performed by χ^2^ test.

**Table 2:** Percentage of adherence to the MDP according to period of interview and selected socio-demographic and lifestyle variables.

	Period of Interview (2009–2010)		Period of Interview (1999–2000)		
Socio-Demographic and Lifestyle Variables	Mean	SD	Mean	SD	*p*-Value
Sex					
Male	44.86	7.71	42.13	7.74	0.001
Female	44.99	7.11	43.39	7.13	0.001
Age group					
16–25	42.91	7.55	42.70	7.66	0.650
26–45	45.19	7.34	42.73	7.36	0.001
46–65	48.39	6.95	43.99	6.89	0.001
Marital status					
Divorced	45.56	7.49	43.45	7.53	0.317
Widowed	43.79	8.71	43.59	8.62	0.993
Married	46.39	6.53	43.26	6.52	0.001
Single	44.01	7.74	42.89	7.77	0.067
Place of birth					
Balearic Islands	44.09	7.14	42.87	7.17	0.001
Spanish East coast	47.43	8.03	43.96	8.23	0.020
Other Spanish regions	48.38	7.44	43.96	7.35	0.011
Other country	45.72	7.94	42.72	8.46	0.028
Educational level					
Low	44.74	7.69	44.04	7.69	0.142
Medium	44.60	7.38	43.12	7.47	0.001
High	45.40	7.57	43.11	7.62	0.017
Socio-economic status					
Low occupational level	44.29	8.03	42.63	8.27	0.088
Medium occupational level	45.29	6.40	43.15	6.38	0.001
High occupational level	45.98	6.94	42.83	6.94	0.227
Physical activity levels					
Sedentary	43.98	8.19	43.42	8.31	0.319
Light	45.66	7.98	44.70	8.09	0.259
Active	45.69	7.70	46.06	7.78	0.332
Smoking habit					
Yes	44.50	8.39	46.42	8.44	0.493
Occasionally	45.45	7.83	44.70	7.55	0.162
No	44.90	8.09	46.06	8.08	0.961

Educational level is grouped according to years and type of education: low (6 years of education); medium (6–12 years of education); high (>12 years of education). Socio-economic level is based on the occupation of the head of household. Physical activity level was determined according to the level of exercise reported by the interviewee during their free time. Smoking habit was classified as: no (never); yes (>1 cigarette/day); occasionally (<1 cigarette/day). Data are expressed as mean (SD); statistical analysis was performed by ANCOVA, trend analysis, adjusted by sex and age.

**Table 3:** Changes (2000–2010) in highest adherent individuals to the MDP (quartile 4) according to selected socio-demographic and lifestyle variables.

	Highest Adherence to the MDP (Quartile 4 vs*.* Quartiles 1 + 2 + 3)	
Socio-Demographic and Lifestyle Variables	Period of Interview (2009–2010)	Period of Interview (1999–2000)
Sex		
Male	1.54 (0.96–2.46)	1.00 (ref.)
Female	0.85 (0.65–1.11)	1.00 (ref.)
Age group		
16–25	0.74 (0.49–1.11)	1.00 (ref.)
26–45	1.15 (0.81–1.65)	1.00 (ref.)
46–65	1.64 (1.07–2.50) *	1.00 (ref.)
Marital status		
Divorced	1.00 (0.37–2.68)	1.00 (ref.)
Widowed	1.11 (0.20–6.11)	1.00 (ref.)
Married	1.30 (0.93–1.82)	1.00 (ref.)
Single	0.91 (0.65–1.28)	1.00 (ref.)
Place of birth		
Balearic Islands	0.89 (0.67–1.17)	1.00 (ref.)
Spanish East coast	1.22 (0.61–2.45)	1.00 (ref.)
Other Spanish regions	1.16 (0.63–2.14)	1.00 (ref.)
Other country	1.70 (0.68–4.29)	1.00 (ref.)
Educational level		
Low	0.92 (0.47–1.80)	1.00 (ref.)
Medium	0.82 (0.61–1.10)	1.00 (ref.)
High	1.45 (0.84–2.52)	1.00 (ref.)
Socio-economic status		
Low occupational level	0.53 (0.34–0.83) **	1.00 (ref.)
Medium occupational level	1.41 (0.88–2.26)	1.00 (ref.)
High occupational level	0.80 (0.44–1.45)	1.00 (ref.)
Physical activity levels		
Sedentary	0.72 (0.44–1.16)	1.00 (ref.)
Light	0.51 (0.35–0.75) **	1.00 (ref.)
Active	1.17 (0.12–11.4)	1.00 (ref.)
Smoking habit		
Yes	0.23 (0.12–0.45) ***	1.00 (ref.)
Occasionally	1.01 (0.54–1.90)	1.00 (ref.)
No	0.49 (0.11–2.19)	1.00 (ref.)

Educational level is grouped according to years and type of education: low (6 years of education); medium (6–12 years of education); high (>12 years of education). Socio-economic level is based on the occupation of the head of household. Physical activity level was determined according to the level of exercise reported by the interviewee during their free time. Smoking habit was classified as: no (never); yes (>1 cigarette/day); occasionally (<1 cigarette/day). Data are expressed as odds ratio (95% confidence interval). Binary logistic regression analysis comparing the period of interview between highest adherent participants to the MDP (fourth quartile) and the other participants as reference value (quartiles 1 + 2 + 3) in selected socio-demographic and lifestyle variables; * *p* < 0.05, ** *p* < 0.001, *** *p* < 0.001.
